# Alcohol Is Food

**Published:** 1870-07

**Authors:** 


### ALCOHOL IS FOOD.

Liquor fattens; whiskey is a good tonic; bitters aid digestion. These are statements made every day with considerable confidence, and in a manner well calculated to impose on a certain class of minds; hence, it is well that the friends of true temperance should have at hand the weapons of their warfare against the liquor traffic.

If alcohol is food, why not give it to our horses?

If liquor fattens, why not give it to our beef cattle, our turkeys, and our pigs; a good dram of it night and morning?

If whiskey is a good tonic, that is, gives a good appetite, why is it that so many whiskey drinkers, the men who are always full and never empty, eat so little; and on the contrary almost live on whiskey? Give them plenty of whiskey and they want nothing else but leisure to drink it.

If "bitters" aid digestion, why is it that those who take them all the time, are never well?

But suppose that in some cases spirits do fatten, it is a watery fat; gives no strength but increases the inability to work, and the susceptibility to all prevalent diseases. In cholera and all epidemics the liquor drinkers are the first to die.

If liquor fattens, why is it that we see so many spindle-shanked drunkards? Whiskey drinkers are often long, lank, and lean, with so little flesh on their bones, that the skin seems almost to cling to them, and so tottering are they in their step that the wind is ready to blow them away at any moment, and so shaky do they become in the end, that it requires all the strength and steadiness of both hands to carry a glass of grog to their lips.

